# Investigating brain dysfunction in neuropathic pain with MRI

**DOI:** 10.1093/braincomms/fcaf196

**Published:** 2025-05-29

**Authors:** Roland Peyron, Siloé Corvin, Camille Fauchon, Isabelle Faillenot

**Affiliations:** INSERM U1028, NEUROPAIN, UJM, UCBL, CNRS UMR5292, Centre de Recherche en Neurosciences de Lyon, F-42023, Saint-Etienne, France; UJM, INSERM, UCBL, CNRS, CRNL U1028, NEUROPAIN team, Saint-Etienne F-42023, France; Department of Neurology & Pain Center, University Hospital, CHU, Saint-Etienne F-42055, France; INSERM U1028, NEUROPAIN, UJM, UCBL, CNRS UMR5292, Centre de Recherche en Neurosciences de Lyon, F-42023, Saint-Etienne, France; INSERM U1028, NEUROPAIN, UJM, UCBL, CNRS UMR5292, Centre de Recherche en Neurosciences de Lyon, F-42023, Saint-Etienne, France; UJM, INSERM, UCBL, CNRS, CRNL U1028, NEUROPAIN team, Saint-Etienne F-42023, France; UJM, INSERM, UCBL, CNRS, CRNL U1028, NEUROPAIN team, Saint-Etienne F-42023, France

**Keywords:** neuropathic pain, central pain, allodynia, insula, SII

## Abstract

Neuropathic pain is a severe chronic disease following nervous system lesions. Allodynia is a main symptom of neuropathic pain, and it can be easily triggered by normally innocuous stimuli inside a functional MRI magnet. In this new series of 35 patients (age ranges: 33–82 years old, 14 females, 21 males, peripheral neuropathic pain: 4, central neuropathic pain: 31), we investigated mechanical dynamic and thermal cold allodynia. Patients were enrolled for the study if allodynia was intense on one part of the body and very slight—or absent—on another part of the body. Allodynia was associated mainly with bilateral increases of activity in anterior insular cortices, anterior mid-cingulate cortex, prefrontal cortex and secondary somatosensory cortices. Most of these activities were correlated with the subjective perception of allodynia, and thus, they dealt with abnormal pain perception. Since these patients also had sensory loss in or around the areas of allodynia, we examined the hypothesis of structural abnormalities in brain structures receiving sensory inputs. Secondary somatosensory cortex ipsilateral to pain showed grey matter loss, and there was a correlation between sensory loss and grey matter density in the lateral thalamus contralateral to pain. The allodynic brain activations were found to be influenced by individual variables describing the patients: the inclination of the patients to experience provoked pain—as defined by quantitative sensory testing/laser-evoked potentials—exacerbated secondary somatosensory cortices activations during allodynia, with the possible consequence that excito-toxicity or similar mechanisms could (secondarily) lead to structural abnormalities. Conversely, we found a negative weighting of ongoing pain level on the allodynic responses in contralateral anterior insula, frontal operculum and parts of secondary somatosensory cortices, suggesting that these regions previously engaged in spontaneous pain had limited possibilities to further increase their response in case of allodynia. In this new series of patients, we confirmed that brain areas that are normally not involved during innocuous stimulations became overactive in case of mechanical allodynia. These results suggest that the above-reported areas could be new targets for neuromodulation techniques with the aim to induce pain relief.

## Teaser

Peyron *et al*. reported that only a few brain areas could generate allodynia in patients with neuropathic pain. These areas are anterior insula, anterior mid-cingulate and secondary somatosensory (SII) cortices. Structural and basal abnormalities in SII after a causal lesion may trigger abnormal reorganizations leading to chronic pain.

## Graphical Abstract

**Graphical Abstract:**
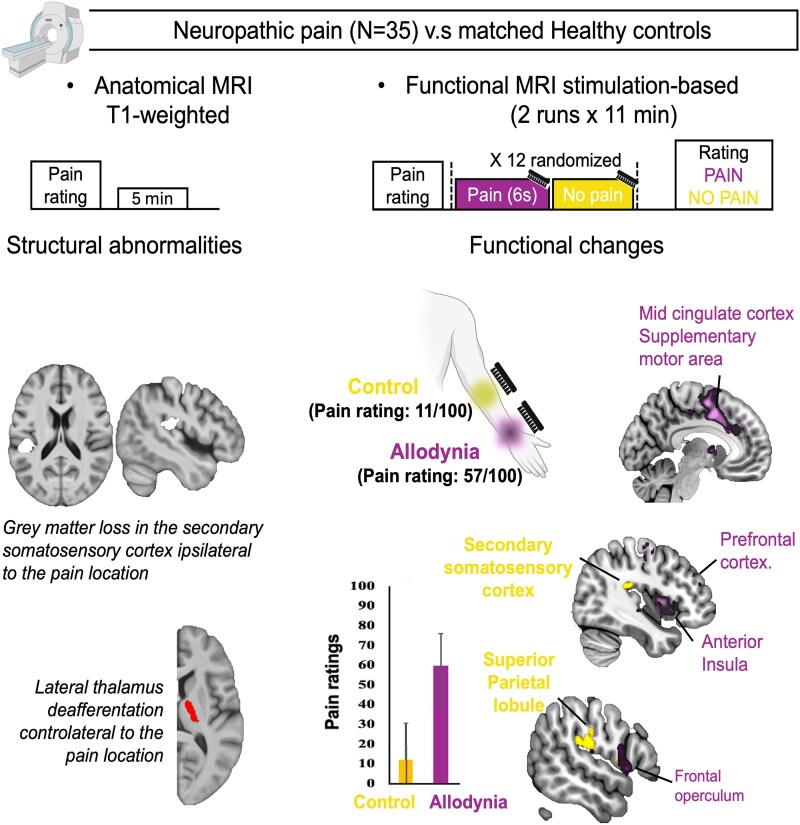

## Introduction

The incidence of neuropathic pain (NP) is ranging between 7% and 10%^1^ in case of stroke, multifocal sclerosis, trauma, spinal cord injury or peripheral lesions, or any other lesion inducing a sensory deficit. Lesion is a necessary condition for neuropathic pain to occur, and it is generally admitted that the lesion must involve the spinothalamic tracts.^2^ At the macroscopic level, the main issue is how the nervous system reorganizes its functions after a lesion. In that sense, it may be seen as a model of reorganizations leading to chronic pain.

Nevertheless, only a little number of previous studies have addressed this issue. Such a high incidence coupled with resistances to usual therapies have prompted many research programmes in spite of which it still remains unknown how brain reorganizations after a spinothalamic lesion may lead to chronic pain.

Previous studies have reported both morphological and functional changes in the thalamus contralateral to pain. Thalamus may therefore appear as a generator for deleterious brain reorganizations in neuropathic pain.^3-5^ Functional abnormalities have also been reported in the operculo-insular cortices during allodynia^6,7^ and in the dorso-lateral-prefrontal cortex (dlPFC) of patients with chronic low back pain.^8,9^

These previously reported abnormalities are difficult to reconcile together except if they reflect different processes: for example, neuropathic pain being systematically associated with sensory loss, spinothalamic hypoesthesia/anaesthesia may be seen as a confounding factor, absent in other kind of pain. Similarly, lesions generating neuropathic pain systematically involve the nociceptive pathways, and thus, loss of inputs is susceptible to influence grey matter density in the brain.

To address these issues, we selected a new large series of patients (*n* = 39) with neuropathic pain in whom allodynia was the main symptom. Abnormal brain responses to allodynic stimulations were investigated using fMRI, provoked pain being easily investigated inside the magnet because it can be triggered on demand. Possible grey matter changes in these patients were also addressed as compared to an age- and sex-matched sample of volunteers. Finally, quantitative influences of sensory loss, and spontaneous ongoing pain—both as a ‘trait’ of NP and as a ‘state’ during the experiment—were also investigated on both structural and functional results.

Our first hypothesis was that lateral thalamic area could present a loss of grey matter density as a function of sensory loss, and that this loss of grey matter could extend to cortical projections, namely the secondary somatosensory (SII) areas. Our second hypothesis was that allodynic stimulation could trigger an amplified response in nociceptive areas such as anterior insula, frontal operculum and medial prefrontal cortices.

## Materials and methods

### Patients and volunteers selection

A new series of 39 patients were selected for the present study based on clinical criteria of a demonstrated neuropathic pain^10^ of peripheral (*n* = 4) or central (*n* = 35) origin.^11^ The presence of a unilateral (mechanical dynamic and/or thermal cold) allodynia was mandatory. Conversely to previous studies,^5,7,12^ we prospectively selected patients with two different levels of allodynic intensity on the same side of the body. In a first body location, allodynia (A condition) was severe (VAS > 5) and in a second location (control or C condition), allodynia was minimal or absent, allowing to contrast directly these two conditions. During the inclusion visit, each patient was tested in two different areas either with a soft brush (made for surgical use, Br) or a with a flat smooth plastic container filled with ice (Co). For reproducibility reasons, the stimuli were the same as those used in previous studies.^5,7,12^ The contact temperature was ambient for the Brush and 1.8 ± 2.4°C for the cold object. Both stimuli were equally rubbed over the skin to check that two distinct level of dynamic mechanical allodynia could be elicited with one of the stimuli.

In these patients, both spontaneous ongoing pain^13^ that may be (or not) associated with allodynia and a variable level of hypaesthesia could be present. Spontaneous paroxysmal pain^13^ may be also addressed in fMRI experiments, but only with very different acquisition paradigms, and thus, this kind of pain was an exclusion criterion in this study.

Computed on a large sample size, this study also investigated grey matter volume changes (structural study). To do so, an age- and sex-matched sample of 27 normal volunteers (often the partner of the patients) was enrolled as a control for the structural study. According to the Declaration of Helsinki, all subjects gave written informed consent for the realization of the fMRI study that had been approved by the national ethics committee (2013-A01791-44 and 2013-A01440-45, Authorizations # 131601B-31 and 140512B-31). Clinical data are presented in Tables 1 and 2, including the tested medications at the time of the study. Overall patients received medications in agreement with French recommendations for the treatment of neuropathic pain^14^ with the adjunction of clonazepam in case of insomnia pain.

**Table 1:** Clinical data: demography, lesions and medications

Patient			Lesion				
#	Age	Sex	Level	Nature	Side	Location	Medications
1	64	f	Nerve endings	Zona	R	Dorsal D6–D8	L P D C
2	47	m	Nerve endings	Trauma	R	Hand trauma	P A D V Clo C (K L)
3	51	m	Nerve endings	Trauma	R	Knee surgery	P (A)
4	41	f	Nerve endings	Trauma	R	Knee surgery	(P G) D K (A) B
5	77	m	Spinal cord	Myelopathy	R/L	Cervical C4–C5	P Clo
6	70	f	Spinal cord	Myelopathy	L	Cervical C6–C7	P Clo
7	65	m	Spinal cord	Myelopathy	R	Cervical C6	P
8	47	f	Spinal cord	Myelitis	R/L	Cervical C4–C5	P
9	82	m	Spinal cord	Myelopathy	R/L	Cervical trauma	P A
10	33	m	Spinal cord	Cavernoma	R/L	Cervical	A P G Clo
11	54	m	Spinal cord	Cavernoma	R/L	Cervical	A (D) G P V K
12	36	f	Spinal cord	MS	R/L	Cervical	P
13	81	m	Spinal cord	Infarct	R	Cervical C5	P A
14	50	m	Spinal cord	Myelopathy	L	Cervical C7	P (A) D Clo (G) K
15	57	m	Spinal cord	Trauma	R/L	Dorsal D12-L1	(P) G
16	51	m	Spinal cord	Myelopathy	L	Cervical C5C6	(G) P (D) V Clo
17	47	m	Brainstem	Infarct	R	Medulla	(P) G A Clo
18	44	m	Brainstem	Infarct	R	Medulla	L G (Clo)
19	39	f	Brainstem	Infarct	L	Medulla	(P)
20	68	f	Brainstem	Infarct	R	Medulla	P G
21	75	f	Thalamus	Infarct	L	Thalamus	P D (L)
22	60	f	Thalamus	Infarct	R	VPL	
23	58	m	Thalamus	Infarct	L	VPL	P (Clo)
24	68	m	Thalamus	Infarct	R	Thalamus	(P A Clo)
25	68	m	Thalamus	Haematoma	R	Lateral and Inferior Thal.	G Clo
26	43	f	Thalamus	Haematoma	R	Thalamic cavernoma	(P C L) Clo (K)
27	64	m	Thal+	Haematoma	R	Thal. insula	(P D) A K B
28	35	m	Thal+	Trauma/haematoma	L	Thal. sub-thal lenticular	P
29	44	f	Thal+	Haematoma	R	Thal. lenticular	
30	57	m	Cortex	Haematoma	L	Insula	P (Clo)
31	64	m	Cortex	Infarct	R	S2 Post insula	Clo
32	37	f	Cortex	Infarct	R	Sylvian	(P D) A
33	75	m	Cortex	Infarct	L	S1 and M1	G (D Clo)
34	41	f	Cortex	Infarct	R	Insula corona radiata	A G P
35	49	f	Cortex	Infarct	L	Posterior insula	P D A (K)

m, male; f, female; Thal, thalamus; Thal+, lesion is mainly in the thalamus with an extension outside thalamic border; MS, multiple sclerosis; R, right; L, left; Thal., thalamus; Post, posterior; VPL, ventral posterolateral nucleus of thalamus; sub-thal, subthalamic nucleus; S1, primary sensory area; M1, motor cortex; S2, secondary sensory area. Medications: G, gabapentin; P, pregabalin; D, duloxetine; V, venlafaxine; A, amitriptyline; L, lidocaine; C, capsaicin; Clo, clonazepam; K, ketamine; B, botulinum toxin; drugs in brackets, not tolerated and/or no efficiency.

**Table 2:** Experiment description for each patient

						Stimulation during MRI experiment			
Patient	Basal pain		QST/LEPs		Type	Allodynia (A)		Control (C)	
#	Side	VAS	SensVar	PainVar		Location	VAS	Location	VAS
1	R	10	13%	100%	B	Thoracic	65	Abdominal	0
2	R	50	0%	29%	B	Hand	80	Forearm	0
3	R	10	45%	67%	B	Leg	45	Thigh	0
4	R	55	20%	25%	C	Leg	80	Foot	0
5	L	10	12%	13%	B	Digit	80	Forearm	0
6	R	10	0%	3%	C	Hand	40	Leg	10
7	R	30	62%	3%	B	Hand	40	Hand/Forearm	0
8	L	40	0%	14%	B	Forearm	80	Foot	0
9	R	70	51%	9%	B	Hand	10	Leg	0
10	L	25	24%	0%	C	Hand	40	Forearm	0
11	R	40	72%	0%	C	Leg (internal)	85	Leg (external)	65
12	L	65	59%	0%	C	Leg	70	Forearm	50
13	L	10	96%	0%	C	Foot	60	Hand	0
14	R	30	MD	MD	B	Forearm	65	Hand	0
15	R	20	70%	0%	B	Thigh	40	Foot	0
16	R	80	55%	0%	B	Forearm	40	Thigh	0
17	L	0	96%	96%	C	Thigh	40	Leg	0
18	L	0	0%	100%	C	Trunk	55	Leg	20
19	R	20	99%	0%	C	Thigh	65	Hand	0
20	L	30	48%	71%	C	Thigh	60	Foot	0
21	R	70	41%	0%	B	Foot/leg	70	Thigh	60
22	L	50	12%	0%	B	Thigh	60	Leg	0
23	R	65	35%	32%	B	Knee area	MD	Thigh	MD
24	L	10	7%	0%	B	Hand	55	Leg	20
25	L	40	100%	0%	C	Thigh	70	Foot	10
26	L	50	64%	61%	C	Hand	35	Leg	15
27	L	5	33%	0%	C	Thigh	30	Leg	0
28	R	25	MD	MD	B	Forearm	80	Thigh	40
29	L	60	78%	74%	B	Foot	60	Leg	30
30	R	70	44%	34%	B	Leg	65	Thigh	0
31	L	25	36%	48%	B	Forearm	80	Thigh	40
32	L	55	44%	67%	B	Leg	65	Foot	0
33	R	40	100%	98%	B	Hand	20	Leg	0
34	L	10	67%	42%	B	Arm	60	Thigh	0
35	R	50	62%	72%	B	Thigh	40	Foot	0

VAS, visual analogue scale for pain rating from 0 (no pain) to 100. Clinical exams: QST, quantitative sensory testing; LEP, laser-evoked potential; SensVar, variable describing hypoesthesia (loss of function. Example: 100% means absence of perception at 50°C, 0% means complete sensory recovery and normal perception threshold); PainVar, variable describing allodynia (pain production. Example: 100% means a maximal allodynia where perception and pain threshold are confounded, 0% means absence of pain perception for 50°C); mechanical allodynia is triggered with B: brush or C: cold; MD, missing data; L, left; R, right.

### Activation study paradigm

**Figure 1:**
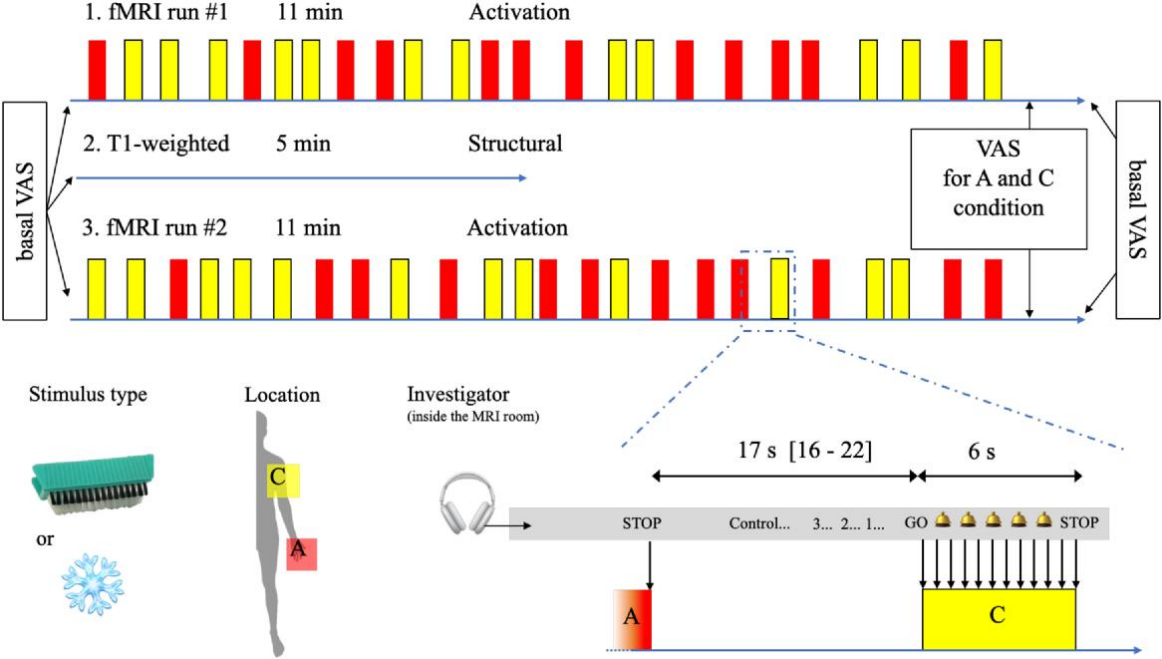
**MRI protocol.** For each patient, two fMRI runs were recorded (sequences #1 and #3). The location of stimulations and the type of stimulus (brush/cold object) were selected with the aim of generating a tolerable mechanical allodynia on one part of the body (A condition) and a comparable non-painful (or a minimally painful) sensation on another part (C condition). During fMRI sequences, A and C stimulations were delivered iteratively and randomly on the two pre-defined sites of the body. Stimulations were delivered by the investigator who wore headset inside the fMRI room and received codified instructions to manage the location and rhythm of the stimulations. Both stimulations were rubbed identically on the skin, with the only difference of the location where it was applied. After each activation sequence, patients indicated the level of their provoked pain intensity on a VAS that was projected on a screen and adjusted by means of +/− buttons. Spontaneous (basal) pain was also evaluated at the beginning and at the end of the fMRI sequences by collecting VAS scores. Sequence #2 was the anatomical T1-weighted MRI (0.9 × 0.9 × 0.9 mm) that was performed also in a group of age- and sex-matched healthy subjects for the structural study. A, allodynic stimulation; C, control stimulation; VAS, visual analogic score; fMRI, functional MRI.

Before starting fMRI runs, the two stimuli described above and the localization where they are to be applied during fMRI were determined with the aim to elicit a high intensity allodynia around 7/10 on VAS (A condition) and no allodynia or, if present, at minimal intensity (control or C condition). The same stimulation was applied on the same hemibody on the same surface but on two different sites for A and C conditions. The site of stimulation was delineated on the skin. During the ‘activation’ runs (see Fig. 1), the investigator was inside the MRI room beside the patient. A warning signal was sent to investigator through a headset 3 s before stimulation, then a GO signal when the stimulation must start and a STOP signal to indicate the end of the stimulations. An audio beep was sent to investigator's headset every 0.5 s from the warning to the stop signal to give the rhythm of the stimulation. Then, the name of the following randomized condition (A or C) was announced to anticipate displacement of the stimulation over the next skin area. Two runs of 24 stimulations (12 for each condition) were recorded. The anatomical sequence was inserted as a resting time between these two runs. Stimulation duration was 6 s with a randomized inter-stimulus interval between 16 and 22 s (mean 17.1 s).

### MRI acquisitions

NeuroImaging was performed on a 3 T Prisma MRI scanner (SIEMENS, Germany) using a 64-channel head coil. Structural volumes were acquired with a MPRAGE sequence using TR = 2 s, TE = 2.19 ms, 0.9 mm isotropic resolution, flip angle 8° resulting in 192 sagittal slices. For the ‘activation study’, two functional runs of 322 EPI volumes were acquired in <11 min with blood oxygen level dependent (BOLD) contrasts, using TR = 1970 ms, TE = 30 ms, grappa 2, 36 axial slices of 3 mm, acquired anterior to posterior, interleaved, without gap, 220 mm field of view and a 74 × 74 matrix, resulting in 3 mm isotropic voxels.

### Image processing

Due to movements during MRI (*n* = 1), artefacts (*n* = 1), lesion impacting normalization processes (*n* = 3) or diffusion of allodynia to contralateral side (*n* = 1), four patients had at least one part of their MRI sequences being removed from the analysis.

Images were prepared with respect to the side of allodynia and the supposed crossed lateralization of brain responses. To do so, raw data of patients with right-sided allodynia (18 patients of 38 patients processed) were left/right flipped. Thus, cerebral hemisphere on the right side of figures was contralateral to the stimulation. Pre-processing was done with different tools, depending on the set of images, fMRIPrep for activation study and SPM12/DARTEL for structural study.

### T1 for structural study

Anatomical scans were segmented and pre-registered using SPM12 segmentation. Then segmented grey matter images were spatially normalized to the MNI space (CAT12 template) using the DARTEL toolbox for voxel-based morphometry (VBM). Registrated segmented images were normalized to the Montreal Neurological Institute (MNI) space, modulated and were finally smoothed with an 8 × 8 × 8 mm^3^ kernel. A mean T1 anatomical image was computed for results localization and visualization purposes.

### BOLD and T1 for activation study

EPI and T1-weigthed images were processed using fMRIPrep 20.2.1,^15^ which is based on Nipype 1.5.1. The T1-weighted (T1) images of 35 patients were corrected for intensity non-uniformity with N4BiasFieldCorrection^16^ and then skull-stripped with a Nipype implementation of the antsBrainExtraction.sh workflow using OASIS30ANTs as target template. Brain tissue segmentation of CSF, white matter (WM) and grey matter (GM) was performed on the brain-extracted T1 using fast (FSL 5.0.9^17^). Volume-based spatial normalization to standard space (MNI152NLin6Asym) was performed through nonlinear registration with antsRegistration (ANTs 2.3.3), using brain-extracted versions of both T1 reference and the T1 template. For each of the two BOLD runs, the following pre-processing was performed. First, a reference volume and its skull-stripped version were generated using a custom method of fMRIPrep. A field map was estimated based on a phase-difference map calculated with a dual-echo GRE sequence, processed with a custom workflow of SDCFlows. Based on the estimated susceptibility distortion, a corrected EPI reference was calculated for a more accurate co-registration with the anatomical reference. The BOLD reference was then co-registered to the T1 reference using bbregister (FreeSurfer) that implements boundary-based registration.^18^ BOLD runs were slice-time corrected using 3dTshift from AFNI 20160207.^19^ The BOLD time-series were resampled onto their original, native space by applying a single, composite transform to correct for head-motion and susceptibility distortions. First, a reference volume and its skull-stripped version were generated.

Automatic removal of motion artefacts using independent component analysis^20^ was performed on the pre-processed BOLD on MNI space time-series and spatial smoothing with an isotropic, Gaussian kernel of 6 mm FWHM (full-width half-maximum). Corresponding ‘non-aggressively’ denoised runs were produced after such smoothing. Several confounding time-series were calculated based on the pre-processed BOLD: framewise displacement (FD), DVARS and three region-wise global signals. All resamplings can be performed with a single interpolation step by composing all the pertinent transformations. Resulting images were checked: runs with FDmean > 0.7 mm were discarded leading to the exclusion of one run in three patients and all run in one patient. ‘Non-aggressive’ denoised images were smoothed with an isotropic, Gaussian kernel of 5 mm FWHM (so that they are globally smoothed at 8 mm). A mask was constructed averaging the 35 GM partition images and binarizing this image with a threshold of 0.1. The normalized T1 images of the 35 patients were averaged to be used for visual inspection of results.

### Statistical analysis

#### Structural study

After exclusion of three outliers by using Check Sample Homogeneity of 3D Data tool available with CAT12 extension, 35 patients (age ranges: 33–82 years old, mean 55.5 ± 14, 14 females, 21 males) and 27 control subjects (age ranges: 30–85 years old, mean 55.6 ± 12.6, 13 females, 14 males, no significant age difference between groups, two-sample *t*-test, *P* = 0.98) were compared using a two-sample *t*-test with sex and total intracranial volume (GM + WM + CSF volumes) as covariates of no-interest. An anatomical explicit mask summarizing all the brain areas involved in sensory and pain processes was defined and included thalami, insulae, SI, parietal and frontal opercula, supramarginal gyri, anterior and middle cingulate cortex and supplementary motor area (SMA). Results were presented with a statistical threshold set at *P* < 0.05 after a family wise error (FWE) multiple comparison correction at the cluster level after a threshold of *P* < 0.001 at a voxel level.

The relationship of these functional and structural abnormalities with the reported intensity of pain and the magnitude of sensory deficit were also investigated.

#### Activation study

Statistical analysis was done with SPM12 in two steps (individual and group) using a mass-univariate approach based on General Linear Models. The design matrix was constructed for each patient with seven regressors per session: control (C) and allodynia (A) conditions convolved with HRF and its derivative, and three confounds: region-wise global signals of CSF and WM and the constant term. These confounds were added to remove residual (non-motion related) structured noise as recommended in Pruim *et al*.^20^ As in our previous studies, we had to consider the presence of brain lesions. With the classical 0.8 threshold recommended by SPM, each lesion would have additively induced an exclusion of its volume from the group analysis. This would have led to unwanted exclusion of several regions, the summation of which would have compromised the whole study. For these reasons, we had checked for the appropriate threshold, allowing to keep the volume of the unitary lesions inside the scope of the group analysis. On the opposite, such a permissive threshold alone would not allow a sensitive analysis because of a too large number of voxels, including those of non-interest outside the brain or inside ventricles, or corpus callosum, for example. Then, to circumvent these limitations, we also applied an exclusive anatomical mask restricting the analysis to regions with a probability of being grey matter > 0.5. The mask constructed with the averaged GM images was used as an explicit mask for the analysis. The cut-off of the high-pass filter was set to 180 s. The GLM was then estimated on the smoothed ‘non-aggressive’ denoised images using classical approach and statistical parametric maps were built for three *t*-test contrasts on HRF-regressors: A, C and A > C. The resulting contrasts images were entered into second-level group analyses. One-sample *t*-tests were carried out for group inferences on C, A and A > C contrasts. Two-sample *t*-tests were applied to groups comparisons [i.e. Brush (Br) versus Cold (Co) stimulations].

For direct comparisons of two conditions or two groups, results were masked with the contrast of the first term alone thresholded at cluster level as described above. For example, the results of A > C were masked with the thresholded image of the A contrast to avoid results due to a bigger deactivation during C than during A condition.

To assess regions where allodynic activations vary proportionally to pain, a ΔVAS covariate—describing differences in pain sensation between A and C conditions—was integrated in a one-sample *t*-test comparing A and C conditions.

Multiple comparisons were FWE-corrected at the voxel level, and at the cluster level after a voxel thresholding at *P* < 0.001. Clusters or voxels were regarded as significant when corrected *P* < 0.05. Each cluster was named relative to the most representative functional region. Each peak was localized on the HCP-MMP1 atlas^21^ for cortical structures or on the BrainNetome atlas^22^ for subcortical structures, on the Morel's atlas^23^ for thalamic nuclei and on the Hammers’ atlas^24^ for insula subdivisions.

#### Clinical variables

Since neuropathic pain is systematically associated with sensory loss in the territory of the pain, we questioned through a quantified sensory variable (sensVar) whether sensory deficits can participate in imaging results. Similarly, due to their lesion, patients may have deficits of nociceptive pain perception (i.e. hypoalgesia) or enhancements of pathological pain (i.e. spontaneous pain or allodynia). This ability/inability to perceive nociceptive pain and/or to produce abnormal pain was quantified with a pain variable (PainVar) describing the pain status. It can be used to question whether pain status can explain any of the imaging results. Both variables were extracted from values of warm and heat pain detection thresholds obtained with quantitative sensory testing (QST) and/or Yap-laser (see details in Corvin *et al*.^25^ and in Supplementary material). SensVar was derived from warm detection thresholds and was expressed in % of sensory loss: for example, SensVar was 0% for a patient with a warm sensation perceived within normal ranges and was 100% for a patient who did not identify as warm a 50°C stimulus (maximal safety range of the QST, TSAPain, Medoc®). PainVar was defined as a ‘trait’ by a personal quantified score of allodynia, describing in each patient how the lowered pain threshold is generally combined with the increase of sensory threshold (i.e. ‘anaesthesia dolorosa’). PainVar was 100% for patients in whom nociceptive and perceptive thresholds were identical (maximal allodynia). PainVar was 0% for patients in whom nociceptive thresholds was not reached at 50°C. Basal VAS (0–10) was a third independent variable, defined as a ‘state’, at rest, describing the pain level at the beginning of the fMRI session, in the absence of any stimulation. Basal VAS evaluated spontaneous ongoing pain in the area where the pain was maximal. Basal VAS was collected a second time at the end of each fMRI run to ensure that the basal pain did not change during the fMRI experiment.

### Correlations between clinical and MRI variables

SensVar was tested against the GM values in areas with GM atrophy, and it was also tested against GM values in pre-defined regions such as thalamus, SII and posterior insulae since these thalamo-cortical areas directly receive sensory inputs from the periphery.

Influences of Basal VAS and PainVar were also tested against areas with GM atrophy. They were also tested against GM values in the brain areas that are known to participate to the ongoing neuropathic pain experience, namely the contralateral thalamus, SII, posterior insulae, anterior insulae bilaterally and anterior subdivision of mid-cingulate cortex (aMCC) that may be involved in amplification of emotional, anticipatory and behavioural processes.

Since these three variables may have influenced the allodynic overactivations, to test our hypotheses, we performed a multiple linear regression (using PSPP 1.6.2) between the three variables and the GM values measured in the following regions of interest (ROIs): seven ROIs were extracted from the A > C contrast, thresholded at a FWE *P* < 10^−4^ voxel level. These are parietal operculum (PO, SII), frontal operculum (FO), anterior insulae, bilaterally and aMCC. Posterior insula ROIs were anatomically defined, bilaterally as well as contralateral thalamic ROIs constructed using MNI atlas of the thalamus^23,26^ with a lateral thalamic ROI including VA, VL, VM, VPI, VPL, VPM and a medial/anterior thalamic ROI including AD, AM, AV, LD, CeM, CL, CM, Hb, MD, MV, Pf, Pv and sPf. Median value of each ROI was extracted separately for each patient and plotted against the three clinical variables in a regression. When a regression was significant (F-test, *P* < 0.05), coefficients (β) of independent variables were tested by a *t*-test. β with *P* < 0.05 were considered as different from 0 and β with *P* < 0.05, were considered as significant.

## Results

### Structural study

Patients (*n* = 35) as compared to normal controls (*n* = 27) had an overall grey matter (GM) loss in SII ipsilateral to pain (OP1 subdivision, *P* < 0.05 cluster FWE corrected).

### fMRI activation study

**Figure 2:**
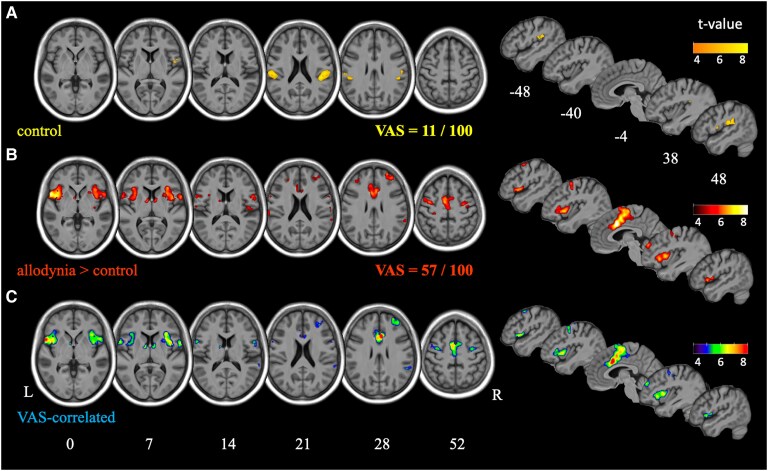
**Activation study.** Brain activations induced by the same stimulation (rubbing the skin) but at different location: (**A**) control stimulation compared to rest was not (or minimally) painful (11/100 on average) and induced limited activations in SII bilaterally, and in superior parietal lobule (SPL, not shown). (**B**) Allodynic stimulation was clearly (57/100 on average) and significantly (*P* < 0.001) more painful than control condition: while stimulations were the same, enhanced activations were found in SII, anterior insulae, frontal operculum, prefrontal cortex, mid-cingulate cortex (MCC) and supplementary motor area (SMA). Please note that the contrast presented here is the contrast of interest A versus C [(A versus Rest) > (C versus Rest)] and not A versus Rest (not shown but results are available in Supplementary Table 1). (**C**) Within the allodynic-related activations presented in **B**, activities correlated with pain intensity (as assessed with VAS scoring) are shown. Results shown in **B** and **C** are very similar, indicating that allodynic-related activations are almost exclusively associated with pain perception. In this figure, activations are the result of one-sample *t*-tests after the most stringent threshold (*P* < 0.05 FWE corrected at the voxel level); in **C**, a covariable VAS has been added to the model. Results are superimposed on the average T1 of the 35 patients included in the study. Colour bars indicate *t*-values. White numbers indicate the MNI coordinates (mm) of the slices. L, left; R, right; VAS, visual analogic score; SII, secondary somatosensory cortex; MNI, Montreal National Institute; FWE, family wise error.

The control condition was associated with increased BOLD signal (Fig. 2A, Table 3 and Supplementary Table 1) in contralateral SI-superior parietal lobule (SPL), bilateral SII, frontal operculum, putamen and premotor cortices (PM and SMA) relative to Rest. While physical characteristics of control and allodynic stimulations were the same except localization of stimuli, allodynic condition induced a significantly higher pain intensity than control condition (57 ± 19 versus 11 ± 19, *P* < 5.e^−14^). Allodynic stimulations induced huge activations encompassing control activations in several brain regions (Fig. 2B, Table 3 and Supplementary Table 1) relative to Rest. Results were not different according to whether rubbing was delivered with a brush or with a cold stimulus (Supplementary Fig. 1 and Supplementary Table 2).

**Table 3:** significant results of the activation study

Region	C (control)	A (allodynia)	A > C	Atlas
**Activations contralateral to stimulations (R)**				
SPL	5L, 7AL	7AL, 7PC, 5L, AIP		HCP
SII	OP1, PFcm, RI	OP1, OP4, PFcm, PFop, RI	OP1, OP4, OP2-3, PFop	HCP
Insula		IG, PoI1, PoI2, MI, AVI PLG, ALG, PSG, MSG, ASG, AIC	PoI1, MI^a^, AVI^a^, AAIC PLG, PSG^a^, MSG^a^, ASG^a^, AIC	HCP Hammers
SI/M1		2	2, 4^a^	HCP
Thalamus			VApc^a^, AV^a^, AM	Morel
FO	FOP1	FOP1, FOP3, FOP4, 6r	FOP4^a^, FOP5^a^, 6r^a^	HCP
dlPFC		9-46d	9-46d^a^, 46^a^	HCP
PM		6d, 6a, FEF	6d, 6a^a^, FEF^a^	HCP
IPL		PFt	PF^a^, PSL^a^	HCP
Caudate Lenticular		Ventral caudate vm and dl putamen, globus pallidus	Dorsal caudate^a^ dl putamen^a^, globus pallidus^a^	BN BN
Others		Sth, V5	Cerebellum^a^, mesencephalon^a^	
**Medial activations (L/R)**				
SMA	6mp	6mp, 6ma, SCEF	6ma, SCEF^a^	HCP
MCC		p32pr, a24pr, 24dd, 24dv	24dv^a^, a32pr, p32pr, 24dd, a24pr^a^, 33pr	HCP
PCC		23c	23c^a^	HCP
**Activations ipsilateral to stimulations (L)**				
SII	OP1, OP4, PFcm, PFop	OP1, OP4, PFop, PFcm	OP4, PFop	HCP
Caudate Lenticular Accumbens		Ventral caudate vm and dl putamen, globus pallidus Accumbens nucleus	Dorsal caudate^a^	BN BN BN
FO		6r	FOP4^a^, FOP5^a^, 6r^a^	HCP
dlPFC		46	9-46d, 9a	HCP
Insula		AVI ASG	MI^a^, AVI PSG, MSG^a^, ASG^a^, AIC	HCP Hammers
PM		6a, 6d, FEF, PEF	6d^a^, FEF^a^, 55b^a^	HCP
Thalamus			VApc^a^	Morel
SI/M1		2, 4	3b^a^, 4^a^	HCP
IPL		PFt		HCP
Others			Cerebellum^a^, V5^a^	

For each contrast and functional region, brain areas with significant voxels were listed. **C** = control and **A** = allodynic conditions compared to Rest; **A** > **C** = (A > Rest) > (C > Rest); note that **C** > **A** did not show any significant difference. ^a^Within the A > C activations, activities that significantly correlated with pain intensity (**Δ**VAS). Labels of the areas come from HCP-MMP1 atlas (HCP)^21^ for cortical structures or BrainNetome atlas (BN)^22^ for the subcortical structures or the Morel's atlas^23^ for thalamic nuclei. For activation in the insula, labels from the Hammers’ atlas^24^ were added. *t*-Tests were applied except for ^a^**Δ**VAS correlation. The threshold to define significant voxels is *P* < 0.05 FWE corrected.

Direct comparison of both conditions [(A versus Rest) > (C versus Rest)] using a stringent statistical threshold showed stronger and bilateral activations in allodynic conditions in the anterior operculo-insular regions, thalami, PM, SI/M1, MCC, dlPFC, SII, cerebellum, contralateral activations in posterior insula and inferior parietal lobule (IPL) and ipsilateral activations in V5 (Fig. 2B, Table 3 and Supplementary Table 1). There was no significant difference in the [(C versus Rest) > (A versus Rest)] comparison.

Within clusters activated by allodynia, voxels having an overactivation [(A versus Rest) > (C versus Rest)] that correlated positively with the increase of VAS (ΔVAS) were mainly located in the anterior operculo-insular and medial frontal regions (Fig. 2C, Table 3).

### Correlations between clinical and MRI variables

**Figure 3:**
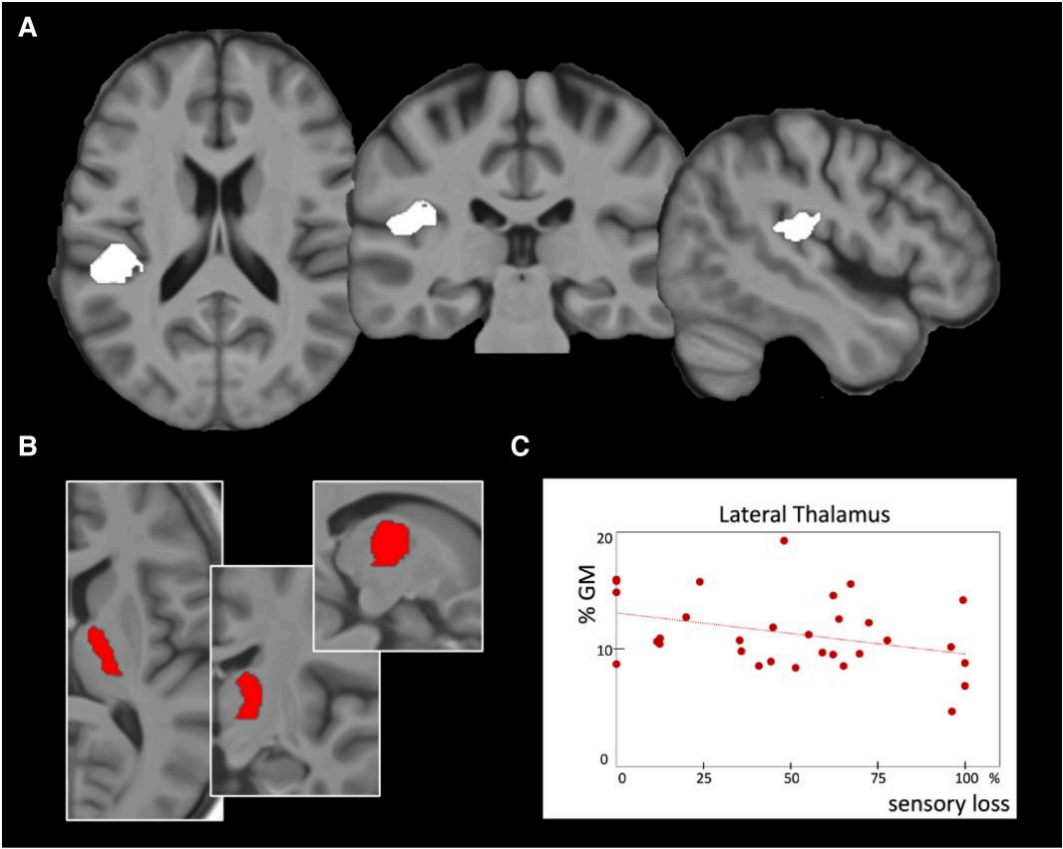
**Structural study.** (**A**) VBM analysis. The comparison of 35 NP patients was made against a sample of 27 age- and sex-matched healthy volunteers. A unique significant cluster of grey matter (GM) loss in SII cortex ipsilateral to pain (two-sample *t*-test, *P* < 0.05, FWE corrected). Three slices are presented: axial *z* = 17; coronal *y* = −29; sagittal *x* = 50 mm in MNI coordinates. (**B**) Only one of the multiple regressions between the mean GM value and clinical variables was significant (F = 4.97, *P* = 0.004): the SensVar variable describing sensory loss was negatively correlated (β = −0.3) with grey matter value in the lateral thalamus ROI, contralateral to pain (red area, *P* = 0.045). Three slices are presented: axial *z* = 9; coronal *y* = −13; sagittal *x* = 14. Results are superimposed on the mean T1 images of the 35 patients. (**C**) Mean GM (*y*-axis) in the lateral thalamus ROI for each patient (expressed in % of the volume of the cluster) are plotted against SensVar that reflects sensory loss (*x*-axis), 0% describing a patient with warm sensations within normal ranges and a 100% value describing a patient who did not identify as warm a 50°C stimulus. Even not considering the other variables (clinical or confounds), figure shows that the more the sensory loss, the less GM volume (*y*-axis) in lateral thalamus. VBM, voxel-based morphometry; SII, secondary somatosensory cortex; ROI, region of interest; SensVar, % of sensory loss; MNI, Montreal National Institute.

The variable describing the loss of sensory function (SensVar) was positively correlated with GM loss (β = −0.3, *t* = −2.1, *P* = 0.045) in the lateral thalamus (F = 5.0, *P* = 0.004), i.e. the more the loss of sensory function, the less lateral thalamic GM (Fig. 3). No other significant influence of SensVar was observed on GM volume, particularly in the ipsilateral SII cortex with GM loss.

The variables describing the propension to pain, i.e. Basal VAS and PainVar, did not correlate with GM volumes in the 10 ROIs tested here.

**Figure 4:**
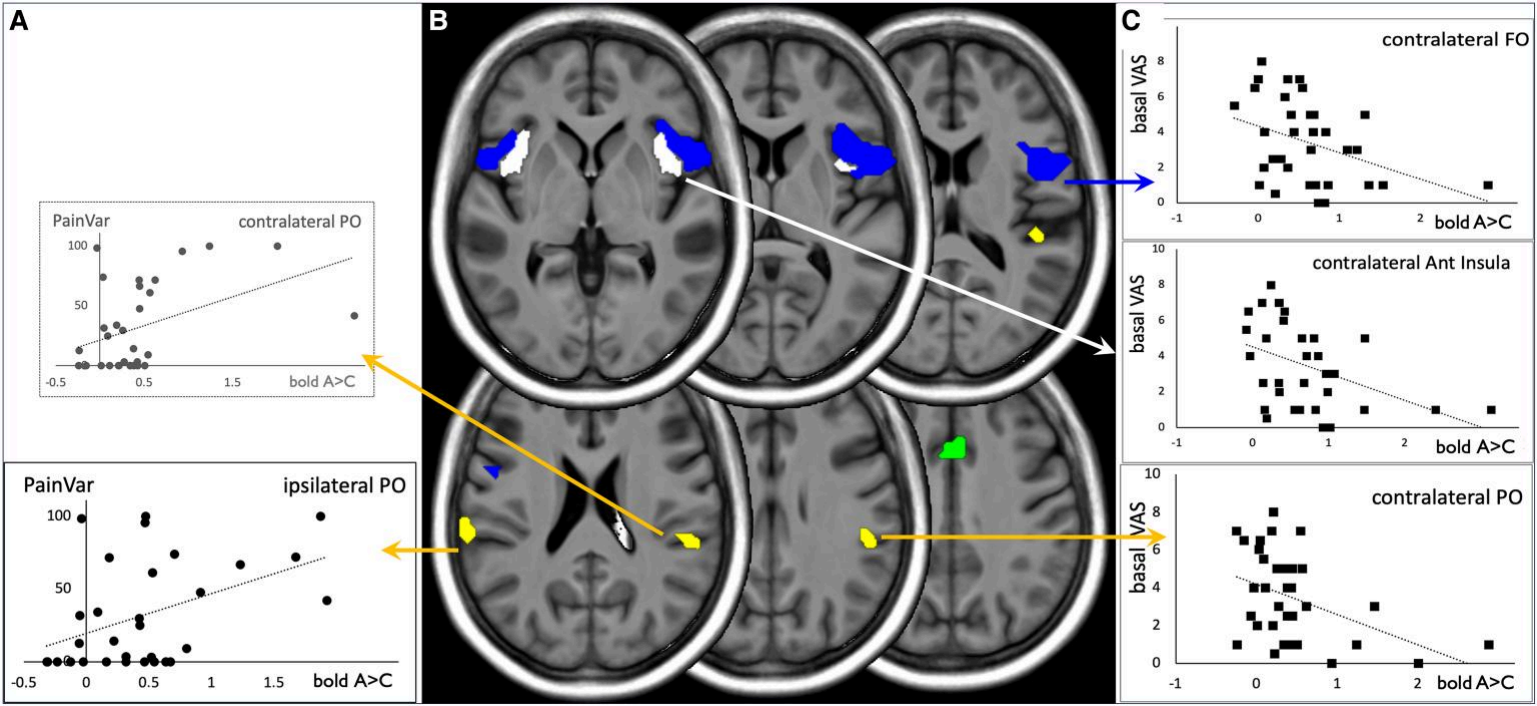
**Effect of pain variables on allodynic overactivations.** The significant cluster A > C representing the comparison A versus C [(A versus Rest) > (C versus Rest)] was segmented in seven anatomical regions: parietal operculum (PO, SII), frontal operculum (FO), anterior insulae (Ant Insula), bilaterally and aMCC . They are presented on the average MRI of the 35 patients (**B**). Mean value of the A > C contrast was extracted for each ROI and for each patient and was tested in a multiple linear regression between these values and clinical variables (SensVar, PainVar and Basal VAS). Among the seven regressions, four were significant (F-test, *P* < 0.05), with at least one variable having a significant β coefficient (*t*-test, *P* < 0.05). In **A**, allodynic overactivations in ipsilateral PO (SII, OP2-3) increased as a function of the ‘trait’ variable PainVar (i.e. the inclination to produce pain). The β coefficient of PainVar in contralateral PO (SII) is near to significance (faded dark box, *P* = 0.06). In **C**, allodynic overactivations were plotted against the ‘state’ variable Basal VAS. Allodynic overactivations were reduced in case of high level of spontaneous pain in contralateral anterior insula, frontal operculum and in PO/SII (OP2-3). VAS, visual analogic score; BOLD, blood oxygen level dependent; A, allodynic stimulation; C, control stimulation; aMCC, anterior mid-cingulate cortex; SensVar, % of sensory loss; PainVar, % of allodynia.

The ‘trait’ variable describing the nociceptive function (PainVar) was positively correlated with the strength of the allodynic BOLD response in SII ipsilateral to pain (β = 0.4, *t* = 2.2, *P* = 0.04) with a trend for SII contralateral to pain (β = 0.3, *t* = 2.0, *P* = 0.06). In these areas, BOLD signal increase was enhanced in patients with high PainVar, meaning that a clinical propension to produce pain is associated with high activations in SII (Fig. 4). The variable describing a ‘state’ of basal (spontaneous) pain during the experiment (Basal VAS) was inversely correlated with the strength of the allodynic BOLD response in contralateral anterior insula (β = −0.4, *t* = −2.6, *P* = 0.02), frontal operculum (β = −0.4, *t* = −2.3, *P* = 0.03) and in restricted subdivisions of SII (OP2-3, β = −0.4, *t* = −2.5, *P* = 0.02). In these areas, allodynic overactivation was significantly reduced in case of high level of spontaneous pain state during the fMRI experiment (Fig. 4).

## Discussion

The finding of a limited number of brain regions in which a normal innocuous sensation was interpreted as a painful one was a main advance for localization of abnormal processes in NP. Anterior insula, frontal operculum, parietal operculum including SII, and medial frontal cortices including anterior MCC and SMA were the main regions in which the activity was abnormally enhanced in the allodynic condition. Only a subgroup of these activations correlated with the intensity of pain, suggesting that some of the reorganizations depended on pain intensity but that some others did not. The anterior operculo-insular cortices and medial and lateral frontal areas correlated with pain intensity but not SII, bilaterally. This observation led to the conclusion that abnormally enhanced activations in SII bilaterally could be necessary to generate allodynia in a first step, but that intensity of pain may be encoded elsewhere in the further steps of the process. The observed overactivations fit with the reported localization of nociceptive processes within the insular cortices: the MSG and ASG subdivisions have been consistently described as crucial for nociceptive processes, particularly in studies comparing supra versus infra-threshold thermal stimulations.^27^ Dissociations between sensory analysis of stimulations and their identification as painful or non-painful have been previously reported in physiological pain.^28-33^ At fixed-energy, laser stimuli were perceived as acute pinprick-heat sensations, but their intensity may vary a lot from a pulse to another, and the corresponding difference was associated with shortened latencies in anterior operculo-insular cortices. More precisely, MSG and ASG could be seen as the two main subdivisions covariating with the intensity of pain sensation. Within the operculo-insular cortex, the anterior subdivisions could be involved in the encoding of pain intensity while the posterior part joined with SII could elaborate the perceptive aspects and discrimination between different stimuli.^27,29,33,34^ In agreement with these segregations of functions in the operculo-insular cortex, patients with lesions in these areas have been reported^35^: they had a very intriguing dissociation between persistent abilities for discrimination of stimuli including pain, but abolished emotional and withdrawal behaviours. Despite the absence of sensory deficit, these patients with asymbolia for pain were unconcerned by pain. Our findings perfectly fit with these previous physiology and lesion reports. From a cytoarchitectonic point of view,^36-38^ this activity mainly concerns agranular and dysgranular cortices. Such an anterior migration of activation from a posterior granular to an anterior dysgranular–agranular cortex has also been reported in two previous series of patients,^5,7^ and this makes sense to consider this anterior migration of the response as significant for the pathophysiological process and for the abnormal perception of pain that refers to allodynia.

Overactivations in the allodynic condition and in correlation with the perceived pain also concerned the aMCC. The contribution of this area to pain perception per se may however be questioned, because of several robust confounds: aMCC is typically involved in attentional processes, particularly attentional shift,^39-41^ but it is also participating with SMA in withdrawal and escape behaviours.^42,43^ These functions are typically not independent from pain intensity scoring since the more the patient has pain, the more he pays attention to what happens (what other groups have qualified as saliency) and the more he tries to escape to the pain. These combined aspects suggest a high probability for aMCC activation to drive attentional functions. Inability to disengage from the perception may also support aMCC results. Although the study was not built to control these confounds, all of them have been observed during the fMRI experiment and reported by patients. In addition, although a few per-operative recordings have identified ‘pain-related’ responses in neurons of MCC,^40,44^ these awakened patients were similarly exposed to the same attentional and motor confounds as our patients. In addition, it has been shown that activities in the cingulate cortex could precede nociceptive stimulation and even determine how the stimulation will be perceived.^45^ As a stronger argument, in large series of direct brain stimulation, a pain sensation has never been elicited by any stimulation at MCC or ACC while it was a frequent observation at the operculo-insular cortices.^46,47^ Consequently, it seems likely that the anterior operculo-insular activities reported here are the most important ones regarding pain production. This finding is important to prompt new neuromodulations techniques targeting this area with the aim to relieve NP. In this perspective, replication of findings is important: most of the allodynic brain activations reported here had been reported previously in patients with neuropathic pain^4-7,12,48,49^ including a crucial role of the thalamic response with an over-reactivity in addition to basal abnormalities.^4,5,12^ An exhaustive list of cortical projections involved in the allodynic process and in chronic pain states includes SII, insula, prefrontal cortex and anterior MCC, bilaterally.^4-8,12,28,48-50^ However, these previous studies were limited by their small sample sizes and their impossibility of direct comparisons between conditions to characterize precisely the allodynic processes. In this new series, we circumvent these two limitations with the largest sample size ever published, and with a different imaging design. In previous series, allodynia was compared to a control stimulation that was applied on the mirror contralateral side. In that case, the control condition was strictly non-painful and in respect with somatotopy but the brain responses to these two stimulations had to be compared while addressing to different hemispheres. The present study has been designed to apply both stimulations on the same side of the body, allowing a direct comparison. These constraints have led to several exclusions of patients with a homogenous distribution of allodynia on their whole hemibody. The present study may therefore present a selection bias with exclusion of patients having extended neuropathic pain and favouring those with neuropathic pain on restricted areas of skin. In other patients, a skin area with minimal (or no) allodynia was found in a place that was not accessible inside the magnet. This has led to additional exclusions. Despite these technical limitations and the fact that allodynic and control conditions were not strictly comparable in terms of somatotopy, we succeeded in recording for all patients, a large gradient of pain intensity between A and C conditions. We assume these choices since pain research is almost familiar with non-somatotopic processes and since the most somatotopic of sensory area in the brain—S1—is an area that is inconsistently activated in pain studies. We also assume that what we report here is not a clear-cut contrast between allodynia and a control condition without pain but that we almost investigate a high and an extremely low—or absent—level of allodynia on the same side of the body.

GM volume in lateral thalamus is proportional to sensory loss on the contralateral side. This finding is consistent with anatomy since lateral thalamus receives the crossed sensory inputs from the periphery. It is also consistent with pathophysiology since neuropathic pain is always associated with thermal sensory abnormalities, and thus, this may be the starting point of other disorders reported here. The cortical areas where sensory inputs project in ipsilateral SII and posterior insula, although tested with *a priori* hypothesis, did not show similar anatomic changes or correlation with sensory loss. This may be at the detection limits of the technique. However, in that case, it is difficult to explain that SII ipsilateral to pain may show significant structural changes as compared to controls. From lateral thalamus, cortical projections are mainly on the same hemisphere. Pain conditions, on the opposite, have always shown bilateral activations in SII.^49,51-53^ The structural abnormalities reported here in SII are lateralized ipsilaterally to pain, and thus, they may have something to deal with pain perception more than with reception of sensory inputs from the periphery. Accordingly, the activations of SII during allodynia were the higher the patients had an allodynic ‘trait’ on the independent variable PainVar (Fig. 4). These results suggest an enhanced allodynic brain response in SII in a first step of amplification of the allodynic process. Structural lesions observed here in SII ipsilateral to pain could be viewed as consequences of these abnormal reorganizations, possibly because of an imbalance in the spinothalamic system and its cortical projections in SII that are known to be consistently bilaterally recruited in contexts of both physiological and neuropathic pain.^49^ One possibility for interpreting these results would be that the functional and the structural changes reported in SII could upregulate the nociceptive system by sending (inappropriately) a nociceptive signal to second or third order areas such as anterior operculo-insular cortices and aMCC in which there are clear evidence of overactivations related to pain intensities, as soon as allodynia is elicited (see Fig. 2C).

The investigations of the concomitant hypaesthesia and their respective structural consequences provided limited results. The brain abnormalities associated with spontaneous ongoing pain were more difficult to interpret than those obtained with provoked pain. We can elaborate the following hypothesis: spinothalamic hypaesthesia is the main deficit in patients with neuropathic pain. The only consequence measured here was a proportional loss of grey matter in the lateral thalamus, in agreement with anatomy, lateral thalamus being a sensory relay on pain pathways. The second main feature in these patients is the production of pain. The variable describing the propension of these patients to produce pain was associated with increased responsiveness of BOLD signal to allodynic stimulation in SII cortices particularly in the side ipsilateral to pain. Do these abnormalities in the long-term drive to excito-toxicity and structural changes in ipsilateral SII? This hypothesis cannot be proved but only suggested based on our results. Finally, the pain state defined as the Basal VAS variable at the time of the imaging experiment showed that a strong basal pain minimized the contralateral allodynic BOLD responses in the crucial areas, namely SII and fronto-insular cortices. These results showed that both pain ‘trait’ and basal pain ‘state’ may influence responses to allodynia and possibly lead to structural changes in crucial areas such as SII, through excito-toxicity or other mechanisms.

## Supplementary Material

fcaf196_Supplementary_Data

## Data Availability

Raw data can be obtained on request to the authors, only in the case of a collaboration project. SPM batches and statistical results of each patient of the activation study and of the group analysis of the structural study are available on a Zenodo repository (DOI 10.5281/zenodo.14178736).
