# Supplementary material for: Investigating brain dysfunction in neuropathic pain with MRI
Source: Brain Commun. 2025 May 29;7(3):fcaf196. doi: 10.1093/braincomms/fcaf196 (PMC12135010; doi:10.1093/braincomms/fcaf196)
Supplement: fcaf196_Supplementary_Data [file fcaf196_supplementary_data.docx]

***Supplementary file - Methods – Clinical variables***

We defined three clinical variables describing sensory loss (SensVar), pain susceptibility (PainVar), and Basal VAS that is simply appreciating basal spontaneous pain before and after the allodynic experiment.

***Material and methods:***

SensVar and PainVar were extracted from QST measurements performed as a sensory inventory in clinical routine in our pain center. We used a Peltier-based contact thermode (20x20mm) delivering a temperature from 0 to 50°C, controlled by WinTSA (Medoc®) software. Briefly, subjects were lying down in a 22°C room. Each stimulation started at the adaptation temperature (32°C). Measurements systematically included Cold and Warm Detection Thresholds (CDT and WDT), Cold Pain Threshold (CPT), and Heat Pain Thresholds (HPT). Since CDT and CPT were less reliable than WDT and HPT, and since Cold stimuli generally exacerbated allodynia, CDT and CPT were not considered for these variables. WDT was determined using the levels method ^(^*^1^*^)^, with a temperature variation of 2.5, 0.7 and 0.2°C and a rate of change of 0.5°C/s. HPT was determined using the limit method ^(^*^1^*^)^ with a temperature varying linearly from 32°C at a rate of 1°C/s. HPT was the average of 4 measurements obtained as the patients stopped the stimulation by pressing a button when it was perceived as painful. Measurements were performed on a body area with allodynia (WDT/HPTpatho), and on a mirror position on the contralateral side (WDT/HPThealthy, hand, n=11, foot, n=4, thigh, n=4), which is the more sensitive way to detect sensory deficits ^(2)^. For patients with bilateral deficits (due to medullary trauma or multiple sclerosis), WDT/HPThealthy (hand, n=6, foot, n=2, thigh, n=1) were those obtained as normative values in normal volunteers.

***Calculation of SensVar***

SensVar was a relative variable quantifying the sensory performance in a 0-100% range where 0% meant that patient did not detect warm at 50°C (i.e. maximal temperature of the QST), and 100% meant that patient had a normal WDT as compared to the homologous (normal) site. SensVar was expressed as the following ratio considering WDT measurements on healthy and pathological body side, and the safety limits of WDT experiment (limit=50.1°C):

sensVar = $\frac{WDTpatho-WDThealthy}{limit-WDThealthy}$

In 4 patients in whom allodynia was located in a very small area, distant from areas of QST measurements and/or in case of a poor accuracy of QST measurements, WDT was replaced by Laser Detection Threshold (LDT). Laser Evoked Potentials (LEPs) were recorded in these patients as a clinical routine with a YAP (Yttrium Aluminium Perovskite) laser delivering a 5-ms stimulation from 0 to 3.5 J with a 0.25 gradation. Before recordings, Detection Thresholds to laser beam were measured similarly what was done with QST. In that case, safety limits for laser were 3 J, 3.5 J or 3.75 J for hands/thorax, thighs/legs and feet stimulations, respectively, to avoid skin injuries. Thresholds were expressed in J/5ms.

***Calculation of PainVar***

PainVar was a relative variable describing the inclination of the patients to produce allodynia, in a 0-100% range. PainVar was 0% for patients with identical perceptive to nociceptive ranges on pathological and healthy areas. It was 100% for patients with confounded WDT and HPT:

painVar = 1 - $\frac{\left( HPT-WDT \right)patho}{\left( HPT-WDT \right)healthy}$

1. M. Mücke, H. Cuhls, L. Radbruch, R. Baron, C. Maier, T. Tölle, R.-D. Treede, R. Rolke, Quantitative sensory testing (QST). English version. *Schmerz*, doi: 10.1007/s00482-015-0093-2 (2016).

2. R. Rolke, R. Baron, C. Maier, T. R. Tölle, - D. R. Treede, A. Beyer, A. Binder, N. Birbaumer, F. Birklein, I. C. Bötefür, S. Braune, H. Flor, V. Huge, R. Klug, G. B. Landwehrmeyer, W. Magerl, C. Maihöfner, C. Rolko, C. Schaub, A. Scherens, T. Sprenger, M. Valet, B. Wasserka, Quantitative sensory testing in the German Research Network on Neuropathic Pain (DFNS): Standardized protocol and reference values: *Pain* **123**, 231–243 (2006).

**
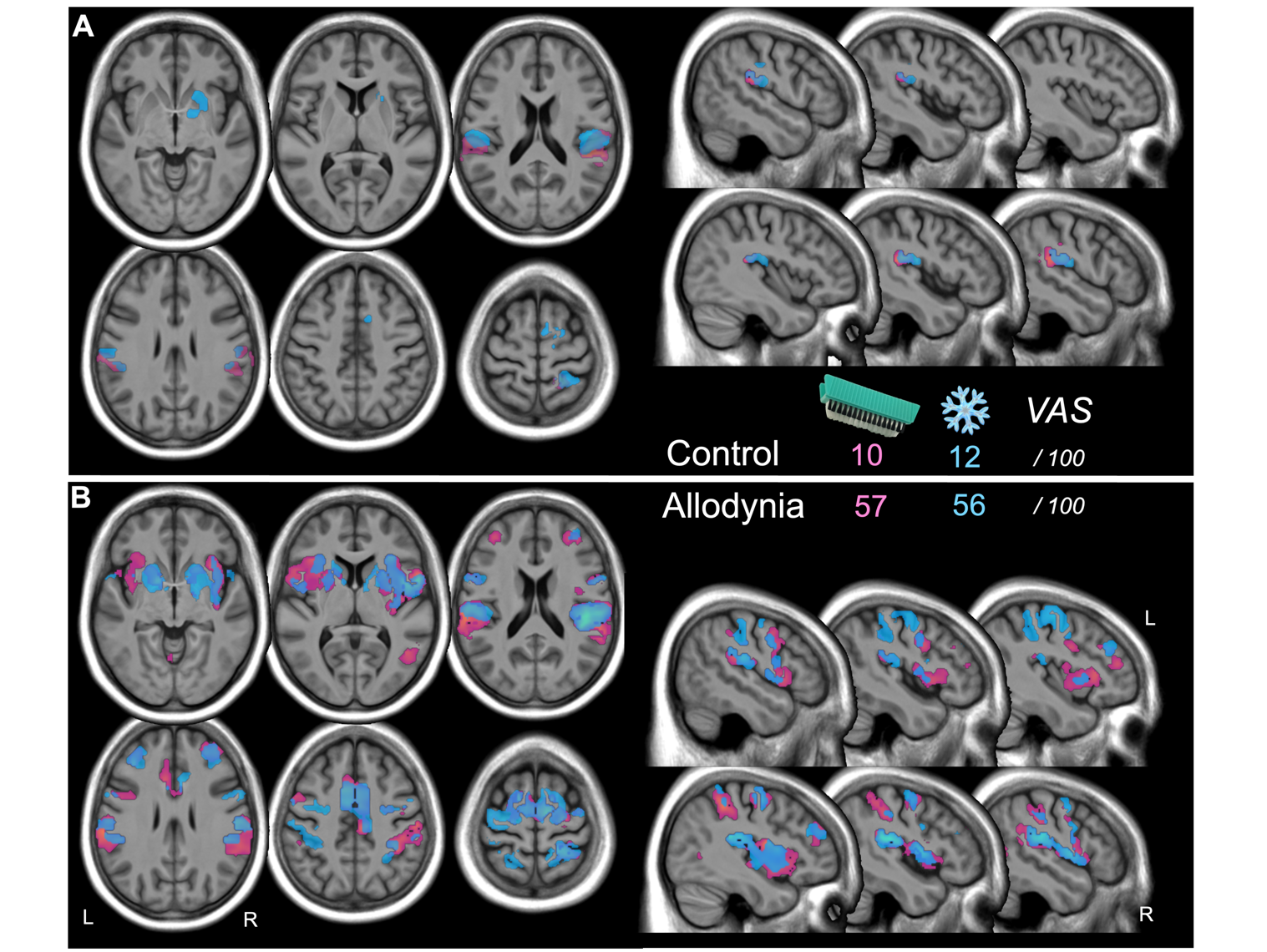
**

**Supplementary Figure 1: Activations to Brush or Cold-mechanical stimulations**

Activations were compared to baseline without stimulation. They were induced by rubbing the skin with a brush (pink, n=20) or with a cold object (blue, n=15). After a one-sample T-test, only voxels with a p<0.001 were kept. Resulting clusters having a size considered as significant (p <0.05 after FWE correction for multiple comparisons) were superimposed on the mean-T1 image. The site of stimulation determines the level of allodynia as indicated on VAS scores.

A. Stimulations were applied on a skin area with minimal or no allodynia (VAS scores 10 and 12/100, respectively).

B. Stimulations were applied on a skin area with high intensity allodynia (VAS scores 57 and 56 /100, respectively).

Please note that activations are the same as those reported in the whole group analysis pooling together the two types of stimuli (Figure 2), and that there is no statistical difference between brush and cold-related activations, neither for the Control nor for the Allodynic condition (two-sample T-Test). L=left; R=right side of the brain.

Supplementary Table 1: Activations during Allodynic and Control stimulations

| **CONTROL**  (voxels where t > 3.4) | | | | | | | **ALLODYNIA** (voxels FWEcorr t > 5.4) | | | | | | | **A > C**  (voxels FWEcorr t > 4.9) | | | | | | **side** |
| --- | --- | --- | --- | --- | --- | --- | --- | --- | --- | --- | --- | --- | --- | --- | --- | --- | --- | --- | --- | --- |
| **CLUSTER** | **chosen PEAKS** | | | | | | **cluster** | | **chosen PEAKS** | | | | | **cluster** | **chosen PEAKS** | | | | |  |
| Size (cm3) | **Areas** | **Coordinates** | | | | **T** | **Size (cm3)** | **Areas** | | **Coordinates** | | | **T** | **Size (cm3)** | **Areas** | **Coordinates** | | | **T** | vs |
| **localization** |  | X | Y | Z | value | | **localization** |  | | X | Y | Z | value | **localization** |  | X | Y | Z | value | pain |
| 12.32 | OP1 | 44 | -27 | 22 | 7.8 | | 22.81 | PoI1 | | 34 | -16 | 4 | 6.3 | 10.06 | AVI | 36 | 22 | -2 | 6.8 |  |
| SII | PFcm | 52 | -22 | 24 | 7.5 | | Insula | MI | | 32 | 14 | 0 | 6.1 | Insula | FOP4 | 44 | 6 | 6 | 7.0 | Controlateral (R) |
| Insula | Ig | 34 | -18 | 12 | 5.7 | | SII | PFcm | | 46 | -26 | 20 | 9.4 | FO | FOP5 | 32 | 26 | 8 | 6.5 |  |
| 3.34 FO | FOP1 | 50 | 2 | 8 | 7.0 | | FO | PFop | | 58 | -18 | 32 | 7.5 |  | 6r | 56 | 8 | 16 | 5.9 |  |
| PM | 6v | 56 | 6 | 34 | 5.0 | | PM | 6r | | 56 | 6 | 18 | 7.1 | 1.92 PM | 6d | 28 | -8 | 68 | 6.0 |  |
|  |  |  |  |  |  | |  | FOP1 | | 46 | 4 | 6 | 8.1 | 0.85 SII | OP2-3 | 46 | -14 | 14 | 5.1 |  |
| 3.41 Putamen | vm put. | 20 | 16 | 0 | 4.9 | | Putamen | vm put. | | 18 | 4 | -2 | 6.8 | Insula | PoI1 | 38 | -16 | -4 | 5.1 |  |
|  |  |  |  |  |  | | IPL | PFt | | 62 | -22 | 38 | 6.1 | 0.70 IPL | PF | 62 | -44 | 38 | 5.1 |  |
| 4.99 | 5L | 18 | -46 | 78 | 8.4 | | 4.97 | 7AL | | 26 | -46 | 64 | 7.6 | 0.02 SPL | 7PC | 38 | -44 | 68 | 5.1 |  |
| SPL | 7AL | 24 | -42 | 68 | 6.8 | | SPL | 5L | | 20 | -46 | 78 | 6.9 |  |  |  |  |  |  |  |
|  |  |  |  |  |  | | 1.42 dlPFC | 9-46d | | 36 | 48 | 24 | 7.3 | 1.69 dlPFC | 9-46d | 28 | 56 | 30 | 6.5 |  |
|  |  |  |  |  |  | | 0.13 S1 | 2 | | 46 | -26 | 42 | 5.6 | 0.01 S1 | 2 | 26 | -42 | 56 | 5.1 |  |
|  |  |  |  |  |  | | 0.03 | STh | | 10 | -10 | -10 | 6.1 | 0.43 Thalamus | VApc/VLa | 8 | -2 | 6 | 5.5 |  |
|  |  |  |  |  |  | | 0.42 V5 | FST | | 46 | -60 | 2 | 6.6 | 1.80 Cerebellum | Lobule VI | 26 | -48 | -22 | 6.6 |  |
| 12.58 | 6mp | 12 | -12 | 70 | 6.3 | | 0.63 PCC | 23c | | 10 | -22 | 44 | 7.4 | 21.58 | SCEF | -8 | -4 | 56 | 7.4 | Midline |
| SMA | 6ma | 14 | 0 | 68 | 5.7 | | 24.64 | 6mp | | 10 | -10 | 68 | 8.4 | SMA | 6ma | 8 | -4 | 70 | 6.2 |  |
|  | 6mp | -14 | -12 | 70 | 5.3 | | SMA | 6ma | | -16 | 4 | 64 | 8.1 | MCC | 24dv | -10 | 4 | 42 | 8.2 |  |
|  | SCEF | -10 | 12 | 46 | 4.3 | |  | SCEF | | -8 | 0 | 50 | 7.9 |  | a32pr | -8 | 30 | 26 | 5.8 |  |
|  |  |  |  |  |  | | MCC | p32pr | | 10 | 6 | 50 | 7.6 |  | 24dd | -10 | -28 | 46 | 5.6 |  |
|  |  |  |  |  |  | |  | 24dd | | 6 | -2 | 52 | 7.5 |  | p32pr | 10 | 18 | 34 | 6.7 |  |
|  |  |  |  |  |  | | M1 | 4 | | 38 | -12 | 54 | 6.6 | 0.26 M1 | 4 | 40 | -12 | 48 | 5.1 | c |
|  |  |  |  |  |  | |  | 4 | | -38 | -12 | 54 | 6.7 | 0.03 M1 | 4 | -26 | -24 | 60 | 5.1 | i |
| 9.07 | OP1 | -52 | -24 | 22 | 8.0 | | 6.72 | OP4 | | -62 | -20 | 22 | 8.3 | 0.39 | PFop | -66 | -24 | 20 | 5.1 |  |
| SII | PFcm | -46 | -32 | 26 | 6.8 | | SII | OP1 | | -52 | -22 | 22 | 7.6 | SII | OP4 | -60 | -12 | 12 | 5.1 | Ipsilateral (L) |
|  |  |  |  |  |  | | IPL | PFt | | -58 | -20 | 32 | 6.0 | 0.33 S1 | 3b | -28 | -28 | 54 | 5.1 |  |
|  |  |  |  |  |  | | S1 | 2 | | -56 | -18 | 36 | 6.2 | 8.79 FO | FOP4 | -54 | 14 | -2 | 8.0 |  |
|  |  |  |  |  |  | |  |  | |  |  |  |  | Insula | MI | -42 | 18 | -2 | 7.4 |  |
| 2.60 | FOP2 | -48 | -2 | 10 | 4.5 | | 0.87 FO | 6r | | -58 | 8 | 20 | 6.4 | 2.45 | 6d | -42 | -2 | 58 | 7.0 |  |
| FO/PM | 6r | -54 | 6 | 32 | 4.8 | | 0.90 PM | PEF | | -56 | 2 | 44 | 6.4 | PM | FEF | -38 | -4 | 50 | 6.3 |  |
|  |  |  |  |  |  | | 0.73 dlPFC | 46 | | -38 | 38 | 30 | 7.2 | 0.44 dlPFC | 9-46d | -24 | 42 | 22 | 5.1 |  |
| 4.44 | v caudate | -12 | 2 | -6 | 5.4 | | 2.80 Insula | AVI | | -30 | 24 | 4 | 5.5 | 0.70 caudate | d caudate | -10 | 0 | 12 | 5.7 |  |
| putamen/ FO | FOP5 | -28 | 26 | 6 | 4.4 | | Lenticular N. | v caudate | | -16 | 6 | -4 | 7.6 | Thalamus | VApc | -8 | 0 | 4 | 6.7 |  |
|  |  |  |  |  |  | |  |  | |  |  |  |  | 0.67 Cerebellum | Lobule VI | -8 | -68 | -16 | 5.1 |  |
|  |  |  |  |  |  | |  |  | |  |  |  |  | 0.02 V5 | MT | -48 | -72 | 2 | 5.1 |  |
|  |  |  |  |  |  | | 0.02 | STh | | -10 | -12 | -10 | 5.9 | 0.01 mesencephalon | | 12 | -26 | -14 | 5.1 |  |

For each contrast, representative peaks were selected within each cluster. Left column: **C** contrast = Control stimulation compared to Rest. Middle column: **A** contrast = Allodynia compared to Rest; Right column: **A > C** = (A > Rest) > (C > Rest); For C contrast, voxels were thresholded at p<0.001 (uncorrected) and clusters at p<0.05 (FWE corrected); for A and A>C, voxels were thresholded at p<0.05 FWE corrected, because allodynic stimulations induced large activations (with a cluster of more than 20 000 voxels with a t>3.3). Labels of the activated areas come from HCP-MMP1 atlas^18^ for cortical structures or BrainNetome atlas^19^ for the central structures or the Morel’s atlas^20^ for thalamic nuclei. Clusters are separated by a black line. Please note that **C > A** did not show any significant difference.

### Supplementary Table 2: Activated regions during Control and mechanical Allodynic stimulations triggered by a skin rubbing with a Brush or with a smooth cold object.

| **contrast** | **CONTROL** Tvox > 3.36 cluster FWE Corr | | | | | | **ALLODYNIA** Tvox > 5.46 voxel FWE Corr | | | | | | **side** |
| --- | --- | --- | --- | --- | --- | --- | --- | --- | --- | --- | --- | --- | --- |
|  | **CLUSTER** | **chosen PEAKS** | | | | | **CLUSTER** | **chosen PEAKS** | | | | |  |
|  | Size (cm3) | Areas | Coordinates | | | T | Size (cm3) | Areas | Coordinates | | | T | vs |
|  | localization |  | X | Y | Z | value | localization |  | X | Y | Z | value | pain |
| **BRUSH (n=20)** | 7.02 | PFcm | 44 | -32 | 22 | 5.7 | 2.28 | PFcm | 46 | -26 | 18 | 6.2 | Controlateral (R) |
|  | SII | PSL | 54 | -34 | 18 | 5.0 | SII | OP1 | 44 | -18 | 16 | 5.8 |  |
|  |  | Pfop | 66 | -24 | 26 | 4.2 | Insula | RI | 42 | -34 | 22 | 6.6 |  |
|  | 2.20 SPL | 5L | 18 | -48 | 78 | 6.4 | 0 .60 | 6r | 58 | 8 | 10 | 6.3 |  |
|  |  |  |  |  |  |  | FO / insula | FOP2 | 38 | 0 | 14 | 5.8 |  |
|  |  |  |  |  |  |  | 0.12 PM | 6a | 36 | -8 | 56 | 5.8 |  |
|  |  |  |  |  |  |  | 0.03 IPL | AIP | 34 | -48 | 52 | 5.7 |  |
|  |  |  |  |  |  |  | 0.58 | 6mp | 10 | -8 | 68 | 6.4 | Midline |
|  |  |  |  |  |  |  | SMA | SCEF | -12 | 12 | 46 | 5.6 |  |
|  |  |  |  |  |  |  | MCC | p24pr | 2 | 2 | 40 | 5.5 |  |
|  | 4.42 | OP1 | -52 | -26 | 22 | 5.4 |  |  |  |  |  |  | Ipsilateral |
|  | SII | PFcm | -48 | -32 | 26 | 5.4 |  |  |  |  |  |  |  |
|  |  | PFop | -62 | -24 | 22 | 5.1 |  |  |  |  |  |  |  |
| **COLD (n=15)** | 4.25 | PFcm | 50 | -24 | 22 | 6.9 | 2.56 | PFcm | 50 | -24 | 22 | 8.3 | Controlateral (R) |
|  | SII | Ig | 36 | -18 | 14 | 4.0 | SII | OP1 | 46 | -21 | 18 | 6.4 |  |
|  |  |  |  |  |  |  | Insula | RI | 42 | -32 | 19 | 6.8 |  |
|  | 7.02 | 5L | 18 | -46 | 78 | 5.7 | 0.41 FO / Insula | FOP1 | 48 | 2 | 6 | 6.4 |  |
|  | SPL | 2 | 20 | -42 | 64 | 5.7 | 1.28 | 2 | 20 | -42 | 64 | 7.5 |  |
|  | 3.21 putamen | vm putamen | 20 | 18 | -2 | 5.3 | SPL | 5L | 18 | -46 | 78 | 7.0 |  |
|  |  | Accumbens | 18 | 2 | -8 | 4.6 | 0.18 Lenticular | vm putamen | 20 | 18 | -2 | 6.1 |  |
|  |  |  |  |  |  |  | 0.01 dlPFC | 9-46d | 36 | 50 | 24 | 5.5 |  |
|  | 2.22 | 6mp | 12 | -14 | 72 | 4.7 | 2.42 | 6mp | 16 | -6 | 68 | 6.2 | Midline |
|  | SMA | SCEF | 8 | 0 | 64 | 4.0 | SMA | SCEF | 8 | -2 | 64 | 5.8 |  |
|  | MCC | p32pr | 12 | 10 | 46 | 3.8 | MCC | P24pr | -6 | 0 | 44 | 6.3 |  |
|  | 4.23 | PFop | -56 | -22 | 22 | 6.5 | 0.46 | OP4 | -60 | -18 | 22 | 6.3 | Ipsilateral (L) |
|  | SII | PFcm | -56 | -28 | 24 | 4.3 | SII | OP1 | -50 | -22 | 22 | 5.9 |  |
|  |  | PFt | -50 | -26 | 34 | 3.7 | 0.14 PM | 6mp | -20 | -18 | 66 | 6.6 |  |
|  |  |  |  |  |  |  | 0.27 caudate | v caudate | -14 | 6 | -4 | 6.5 |  |
|  |  |  |  |  |  |  | 0.01 M1 | 4 | -40 | -12 | 58 | 5.8 |  |

Control (vs Rest) and Allodynia (vs Rest) contrasts for rubbing the skin with a brush at ambient temperature (pink color) and for rubbing the skin with a smooth cold object (blue color). Representative peaks were selected within each cluster.

For Control contrasts, voxels were thresholded at p<0.001 (uncorrected) and clusters at p<0.05 (FWE corrected); For Allodynia contrasts, voxels were thresholded at p<0.05 FWE corrected because allodynic stimulations induced large activations (with a cluster of more than 20 000 voxels with a t>3.3). Labels of the activated areas come from HCP-MMP1 atlas^21^ for cortical structures or BrainNetome atlas^22^ for the subcortical structures.
